# Verapamil Suppresses the Development of Resistance Against Anti-Tuberculosis Drugs in Mycobacteria

**DOI:** 10.3390/ijms262211124

**Published:** 2025-11-17

**Authors:** Kunna Liu, Elise Buitenhek, Coenraad P. Kuijl, Yuval Mulla, Joen Luirink, Dirk Bald

**Affiliations:** 1 Molecular Microbiology Section, A-LIFE and AIMMS, Faculty of Science, Vrije Universiteit Amsterdam, 1081 HZ Amsterdam, The Netherlands; k.liu@vu.nl (K.L.); s.luirink@vu.nl (J.L.); 2 Medical Microbiology and Infection Control (MMI), Amsterdam University Medical Center Location VUmc, 1081 HZ Amsterdam, The Netherlands; c.kuijl@amsterdamumc.nl; 3 Amsterdam Institute for Immunology & Infectious Diseases, Amsterdam University Medical Center Location VUmc, 1081 HZ Amsterdam, The Netherlands

**Keywords:** verapamil, mycobacteria, resistance frequency, efflux pump, DNA repair

## Abstract

The emergence of drug resistance remains a major challenge in the treatment of tuberculosis and other mycobacterial infections. To combat the rise in resistance, strategies that reduce the frequency of resistance mutations are urgently needed. Verapamil is a small-molecule compound that can enhance the potency of companion drugs in combination regimen. Here, we investigate if verapamil can decrease the resistance frequency of antimycobacterial drugs. The results show that verapamil significantly reduces the resistance frequency of multiple antimycobacterial agents, including the DNA gyrase inhibitor moxifloxacin, the protein synthesis inhibitor streptomycin, and the RNA polymerase inhibitor rifampicin in *Mycobacterium smegmatis*. The presence of point mutations in the target was confirmed for moxifloxacin-resistant *M. smegmatis*. Suppression of resistance evolution against moxifloxacin by verapamil was also found in the slow-growing, pathogenic mycobacteria *M. avium* and *M. tuberculosis*. Real-time qPCR analysis in *M. smegmatis* showed that verapamil treatment downregulates the expression of multiple efflux pump genes and upregulates DNA repair genes. These findings suggest that verapamil exerts a dual role by interfering with efflux pump functionality and by reducing the probability of chromosomal mutations. The combination of these properties may underlie the promise of verapamil as adjuvant to enhance the effectiveness of current antimycobacterial chemotherapy.

## 1. Introduction

The emergence of (multi)-drug resistant *Mycobacterium tuberculosis* strains is a major factor interfering with efficient tuberculosis control. According to the WHO Global Tuberculosis Report 2024, approximately 400,000 people developed multi-drug-resistant tuberculosis or tuberculosis resistant to the front-line drug rifampicin. The success rate for treating patients infected with these resistant strains was only 68% in 2023 [1]. In addition, infections by nontuberculous mycobacteria such as *Mycobacterium avium* represent a growing health concern due to resistance to a wide range of antibacterials [2]. For these reasons, it is of high importance to utilize antibacterials effectively and minimize the chance of occurrence of drug resistance.

**Figure 1:**
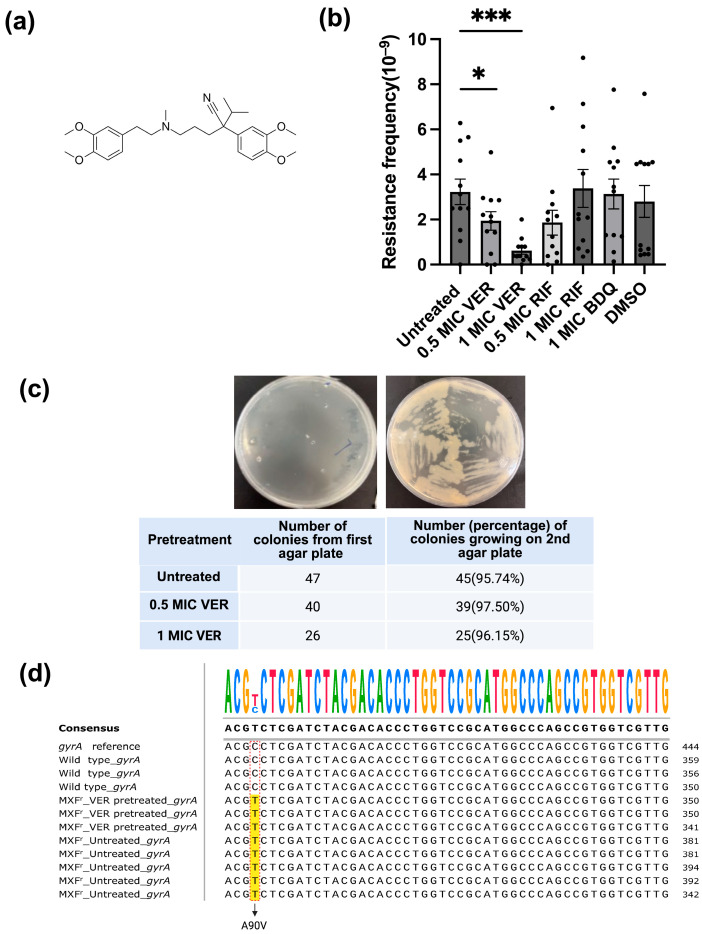
Verapamil reduces moxifloxacin resistance frequency in *M. smegmatis*. (**a**) Structure formula of verapamil. (**b**) Effect of verapamil (VER), bedaquiline (BDQ) and rifampicin (RIF) on the frequency of resistance to moxifloxacin in *M. smegmatis*. The bacteria were pretreated with the drugs (or DMSO/water control) for 48 h and subsequently transferred to agar plates containing moxifloxacin at 2 × MIC. Four biological replicates were used for each sample. Significance for verapamil was calculated vs. untreated (water), and for bedaquiline and rifampicin vs. DMSO, using unpaired one-tailed *t*-tests. Data represent mean ± SEM (*: *p* <  0.05, ***: *p* <  0.001). (**c**) Test for transient resistance versus mutation-based resistance. Bacteria were transferred from colonies on the first agar plate (2x MIC moxifloxacin) to a new agar plate containing moxifloxacin at 2x MIC. A representative streak on the second agar plate (upper panel) and the (relative) number of colonies growing on the second agar plate (lower panel) are shown. (**d**) DNA sequencing of the *gyrA* gene for moxifloxacin-resistant colonies. *gyrA* reference: *gyrA* sequence in NCBI database; wild type_*gyrA*: *M. smegmatis* growing on plate without moxifloxacin; MXF^r^_VER-pretreated_*gyrA*: *M. smegmatis* colonies pretreated with verapamil growing on agar plate with moxifloxacin at 2x MIC; MXF^r^_Untreated_*gyrA*: untreated *M. smegmatis* growing on agar plate with moxifloxacin at 2x MIC. The C → T changes are marked with red rectangles, and the altered nucleotides in moxifloxacin-resistant colonies are highlighted in yellow.

Verapamil is a small-molecule compound (Figure 1a) that can substantially potentiate the antimycobacterial activity of companion drugs in combination treatments in vitro, e.g., as shown for bedaquiline (used for treatment of drug-resistant tuberculosis) [3,4] and for the experimental drug clofazimine [5]. In both *M. tuberculosis*-infected macrophages and a zebrafish model of *M. marinum* infection, verapamil notably reduced tolerance to the front-line drugs isoniazid, ethambutol, and rifampicin, and the second-line drug moxifloxacin [6,7]. A recent clinical trial reported that co-administration with verapamil enhanced the exposure to rifampicin in human pulmonary tuberculosis patients without serious side-effects, suggesting the possibility of verapamil as an adjunctive drug in treatment regimens [8].

Although the mechanism of action of verapamil is subject to discussion, most reports link the action of verapamil to the inactivation of drug efflux pumps [9,10]. It has been demonstrated that verapamil causes enhanced intracellular levels of ethidium bromide in *Mycobacterium smegmatis* [11,12,13] and of a fluorescent derivative of the front-line drug rifampicin in *M. tuberculosis* [14]. Efflux pumps can significantly increase the minimal inhibitory concentration (MIC) for a transported antimycobacterial drug, and are regarded as a determinant for drug resistance in mycobacteria [4,5,6,7,15,16]. Resistance caused by this mechanism typically is of a relatively low level [15,17]. However, low-level resistance caused by efflux pumps can maintain survival of a mycobacterial sub-population, enhancing the chance to develop mutations in the population that lead to clinical levels of resistance, as shown for the front-line anti-tuberculosis drug isoniazid [17].

Intriguingly, a correlation between efflux pump expression levels and development of point mutations leading to antibiotic resistance has been demonstrated in *Escherichia coli* [18,19]. Over-expression of the *acrAB* genes, which encode key components of the resistance–nodulation–division (RND)-type efflux pumps, increased the mutation rate and thereby accelerated the development of resistance against various antibacterials with different mechanisms of action [18]. Inactivation of the *acrB* gene, in turn, decreased the mutation rate and slowed down the development of resistance [18]. In a later study by another group, a similar relation between efflux and development of drug resistance was found in swarming *E. coli* cells [19]. To our knowledge, such a correlation between efflux pump levels, mutation rate, and development of drug resistance has not been reported in other bacteria.

We reasoned that verapamil, thought to interfere with efflux pump functionality, might concomitantly decrease the chance of mutations in the bacterial genome, thereby suppressing the development of drug resistance. Therefore, in the current study, we investigate how treatment of mycobacteria with verapamil influences the resistance frequency for selected first- and second-line anti-tuberculosis drugs.

## 2. Results

### 2.1. Verapamil Suppresses the Development of Resistance in M. smegmatis

We set out to evaluate if verapamil can interfere with the development of resistance in mycobacteria. As a model, we chose the non-pathogenic, fast-growing bacterium *M. smegmatis* [20]. We first evaluated resistance against the second-line anti-tuberculosis drug moxifloxacin, a DNA gyrase inhibitor, as resistance to this drug typically is based on well-defined mutations at specific positions in the *gyrA* and *gyrB* genes [21]. We determined the minimal inhibitory concentration (MIC) of moxifloxacin for the *M. smegmatis mc^2^155* strain in our laboratory to 0.063 μg/mL, similar to values reported in the literature [22,23]. The baseline resistance frequency of *M. smegmatis* against different concentrations (1.5 × MIC-12 × MIC) of moxifloxacin ranged from 4.4 × 10^−9^ to 4 × 10^−11^ (Table S1), consistent with previously reported data [21,24]. To ensure the colony number was within a suitable range for counting (20~300), we used agar plates containing moxifloxacin at a concentration of 2 × MIC to test the effect of verapamil. *M. smegmatis* cultures pretreated with verapamil at 0.5x MIC or 1x MIC for 48 h were plated on agar containing moxifloxacin at 2x MIC. The resistance frequency was calculated as the number of colonies growing on the agar plate with moxifloxacin relative to the number of colonies on antibiotic-free plate. After pretreatment with verapamil at 1x MIC, the number of colonies resistant to moxifloxacin as well as the resistance frequency for moxifloxacin (0.6 ± 0.15 × 10^−9^) was significantly lower as compared to untreated control (3.2 ± 0.56 × 10^−9^) (Figure 1b and Figure S1a,b). In contrast, pretreatment of *M. smegmatis* with rifampicin, which targets RNA polymerase [25], or with bedaquiline, an ATP synthase inhibitor [26,27], did not influence the resistance frequency observed for moxifloxacin (Figure 1b).

Mycobacteria grown in liquid culture are prone to aggregation and clumping, which could lead to artifacts in the determination of the resistance frequency from CFU counts. Therefore, we performed flow cytometry to analyze bacterial cell size, morphology, and state of aggregation. We found no significant differences in morphology (Figure S2a,b) or size distribution (Figure S2c,d) between verapamil-pretreated bacteria and untreated control bacteria. Statistical analysis of the median and mean values indicated comparable cell sizes across different samples (Figure S2e). Thus, decreased bacterial clumping is unlikely to explain the observed verapamil-induced reduction in resistance frequency.

Next, we evaluated if the colonies observed on the agar plates containing moxifloxacin represented transient, phenotypic resistance (e.g., based on transient upregulation of efflux pumps) or resistance based on genetic mutations. We transferred colonies onto a second plate containing moxifloxacin at 2 × MIC and quantified colony numbers. More than 96% of the total selected 113 colonies growing on agar plates containing moxifloxacin at 2 × MIC, either from verapamil-pretreated samples or from untreated control, grew on the second moxifloxacin-containing agar plate as well (Figure 1c), indicating stable genetic mutations rather than transient resistance. Sequencing of the *gyrA* and *gyrB* mutation hotspots revealed that eight tested resistant colonies from both verapamil-pretreated and control samples harbored an identical point mutation (cytosine to thymine) in the *gyrA* gene (Figure 1c). This mutation leads to a change at position 90 in GyrA from alanine to valine (A90V) (Figure 1d), a change frequently observed in moxifloxacin-resistant bacteria [21]. Verapamil apparently does not influence the type of mutations occurring; however, these data confirm the chromosomal mutation nature of the observed resistance.

**Figure 2:**
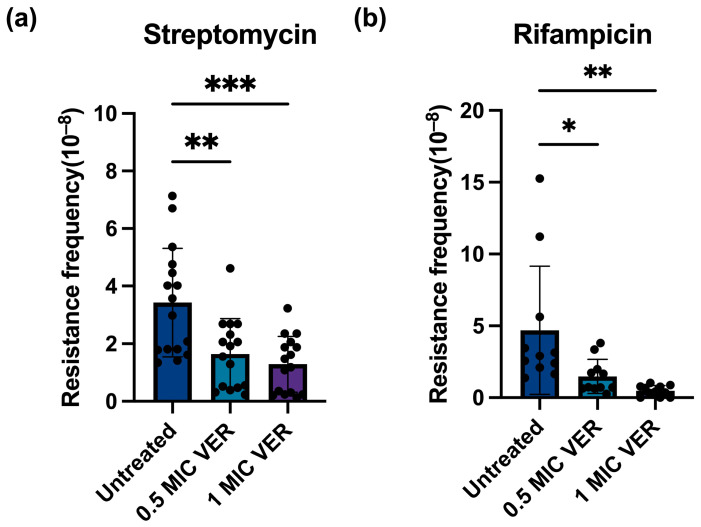
Verapamil pretreatment decreases resistance frequency for streptomycin and rifampin in *M. smegmatis*. (**a**) Effect of verapamil (VER) on the frequency of resistance to streptomycin in *M. smegmatis*. (**b**) Effect of verapamil on the frequency of resistance to rifampicin in *M. smegmatis*. The bacteria were pretreated with 0.5 × MIC or 1 × MIC verapamil for 48 h and subsequently transferred to agar plates containing streptomycin at 2x MIC or rifampicin at 8x MIC. Four biological replicates were used for each sample, data represent mean ± SD. The significance was shown for comparison to the untreated sample, as tested by using unpaired one-tailed *t*-tests (ns: not significant, *: *p* <  0.05, **: *p* <  0.01, ***: *p* <  0.001).

Subsequently, we investigated whether the observed decrease in the resistance frequency caused by verapamil is specific for moxifloxacin or if it represents a broader phenomenon. We tested two additional antibiotics with different mechanisms of action: streptomycin, which inhibits ribosomal protein synthesis [28], and the RNA polymerase inhibitor rifampicin [25]. The same workflow was applied as described above for evaluation of resistance against moxifloxacin. We determined the MIC for rifampicin to 5 μg/mL, and streptomycin to 0.25~0.5 μg/mL, comparable to published values [29,30,31,32,33]. A series of rifampicin and streptomycin concentrations on the agar plate was tested (Table S1). Concentrations of eight-fold MIC and two-fold MIC were chosen for further experimentation with rifampicin and streptomycin, respectively, as these concentrations yielded well countable numbers of colonies. We calculated the resistance frequency for rifampicin to 2.70 × 10^−8^ and for streptomycin to 1.82 × 10^−8^, close to published mutation frequency ranges [34,35]. After verapamil pretreatment, the number of colonies resistant to rifampicin or streptomycin (Figure S1c,d) as well as the resistance frequency for both streptomycin and rifampicin significantly declined (Figure 2). These results suggest that verapamil exhibits a broad effect, interfering with the development of resistance across different classes of antibiotics.

### 2.2. Verapamil Suppresses Resistance to Moxifloxacin in Pathogenic Mycobacteria

**Figure 3:**
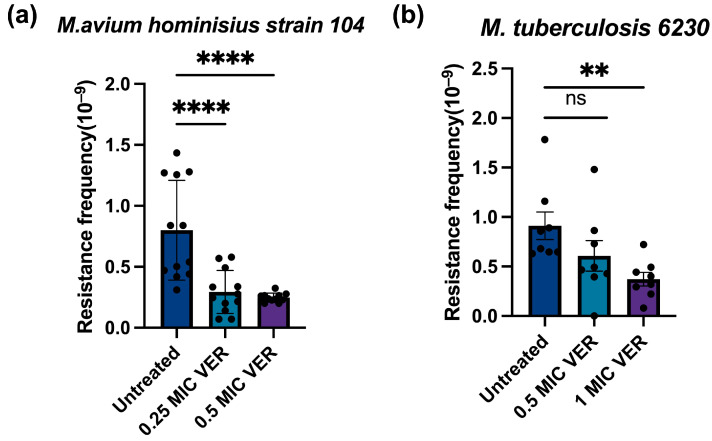
Verapamil pretreatment decreases moxifloxacin resistance frequencies in slow-growing mycobacteria. (**a**) Effect of pretreatment with verapamil (VER) on the frequency of resistance to moxifloxacin in *M.avium hominisius* strain 104. (**b**) Effect of pretreatment with verapamil on the frequency of resistance to moxifloxacin in *M. tuberculosis* mc^2^ 6230. The bacteria were pretreated with verapamil in 0.5 × MIC or 1 × MIC for 48 h and subsequently transferred to agar plates containing moxifloxacin at 2 × MIC. Three biological replicates were used for each sample; data represent mean ± SD. The significance was shown for comparison to the untreated sample, as tested by a one-way ANOVA with Dunnett test for multiple comparisons (ns: not significant, **: *p* <  0.01, ****: *p* <  0.0001).

To assess the clinical relevance of our findings, we investigated whether the effect of verapamil on the development of resistance found above for *M. smegmatis* is an idiosyncratic property of this fast-growing mycobacterium or if it extends to slow-growing and pathogenic mycobacterial species. We tested *M. avium hominisius strain 104*, a slow-growing nontuberculous bacterium causing pulmonary disease [2,36], and *M. tuberculosis* strain mc^2^6230 (*ΔRD1ΔpanCD*) [37], an auxotroph mutant of the pathogenic laboratory strain *M. tuberculosis* H37Rv. As observed above for *M. smegmatis*, we found that pretreatment with verapamil significantly decreased the resistance frequency in *M. avium* and in *M. tuberculosis* (Figure 3a,b). A concentration-dependent reduction in resistant colony numbers was also observed (Figure S3). These results indicate that verapamil can suppress development of antimicrobial resistance in both fast-growing and slow-growing, non-pathogenic and pathogenic mycobacteria. This cross-species efficacy may substantially enhance the potential of verapamil as an adjunctive therapy in clinical settings for combating drug-resistant tuberculosis and other mycobacterial infections.

### 2.3. Decreased Resistance Frequencies Are Not Due to Collateral Sensitivity

**Figure 4:**
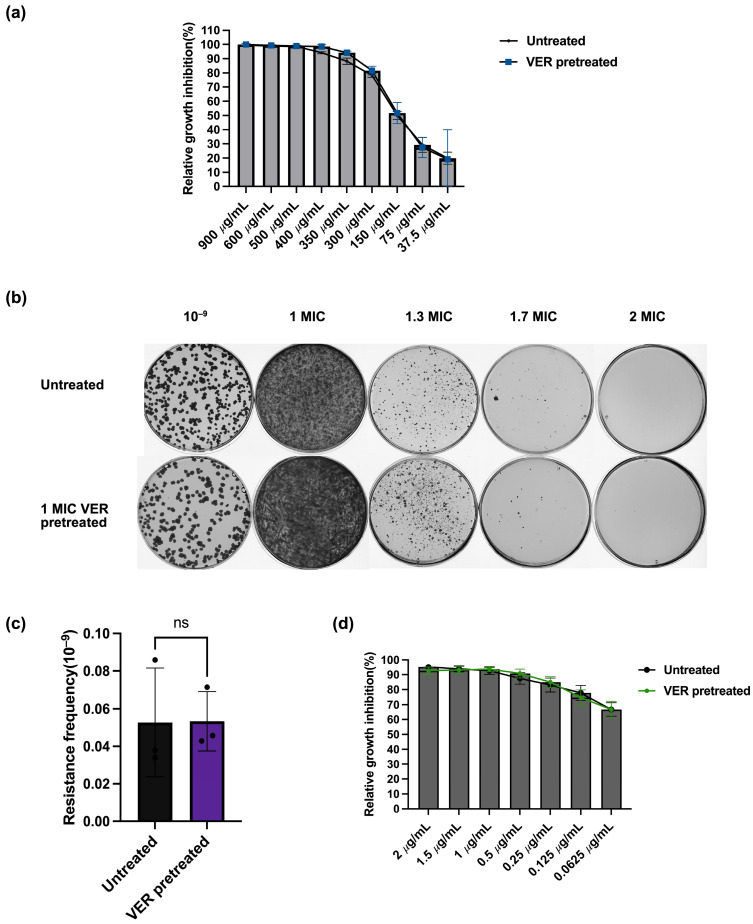
Lack of collateral sensitivity between verapamil and the tested anti-tuberculosis drugs in *M. smegmatis*. (**a**) Relative growth inhibition by verapamil assessed by resazurin fluorescence. Growth of bacteria pretreated with verapamil at 1 × MIC was compared to growth of bacteria without pretreatment. For each verapamil concentration, three biological replicates and for each biological replicate, three technical replicates were performed. Data represent mean ± SD (ns: not significant. (**b**) Growth of *M. smegmatis* pretreated with verapamil (1x MIC) and of untreated control on agar plates with different concentrations of verapamil. (**c**) Resistance frequency of *M. smegmatis* pretreated with verapamil (1x MIC) and of untreated control for growth on agar plates containing verapamil at 1.7 × MIC. Data represent mean ± SD. The significance was shown for comparison to the untreated sample, as tested by using unpaired two-tailed *t*-test (ns: not significant). (**d**) Relative growth inhibition by moxifloxacin assessed by resazurin fluorescence. Growth of bacteria pretreated with verapamil at 1 × MIC was compared to growth of bacteria without pretreatment. For each moxifloxacin concentration, three biological replicates and for each biological replicate, three technical replicates were performed. The significance was shown for comparison to the untreated sample, as tested by using multiple unpaired *t*-test (ns: not significant).

The development of resistance for one drug can lead to enhanced sensitivity for a second, unrelated drug, a phenomenon known as collateral sensitivity. Collateral sensitivity has been found in mycobacteria and can potentially be exploited to optimize drug combination regimen [38]. We investigated if collateral sensitivity might explain the decreased resistance frequency found above upon verapamil pretreatment. During verapamil pretreatment, *M. smegmatis* may develop resistance to verapamil; however, these verapamil-resistant bacteria may be more susceptible to other antibiotics. To test whether verapamil pretreatment induced verapamil resistance, we determined the sensitivity for verapamil before and after the verapamil pretreatment. The result showed no difference in growth inhibition by verapamil between the untreated sample and the verapamil-pretreated sample (Figure 4a). In line with this finding, we observed no difference in colony numbers between the verapamil-pretreated sample and the untreated control bacteria on agar plates containing 1.7 × MIC verapamil, and no colonies were observed on agar plates containing verapamil at 2 × MIC (Figure 4b,c). These results show that verapamil pretreatment did not trigger resistance to verapamil. To further evaluate whether verapamil pretreatment altered bacterial sensitivity to other antibiotics, we tested the growth inhibition of the verapamil-pretreated sample by the three investigated antibiotics. The observed susceptibility did not differ from the susceptibility determined for the untreated controls (Figure 4d and Figure S4). Taken together, it seems highly unlikely that the observed decrease in resistance frequency after verapamil pretreatment is due to collateral sensitivity with the tested antibacterials and instead is likely caused by a decrease in mutation rate.

### 2.4. Verapamil Modulates the Expression of Genes Encoding Efflux Pumps and DNA Repair Enzymes

**Figure 5:**
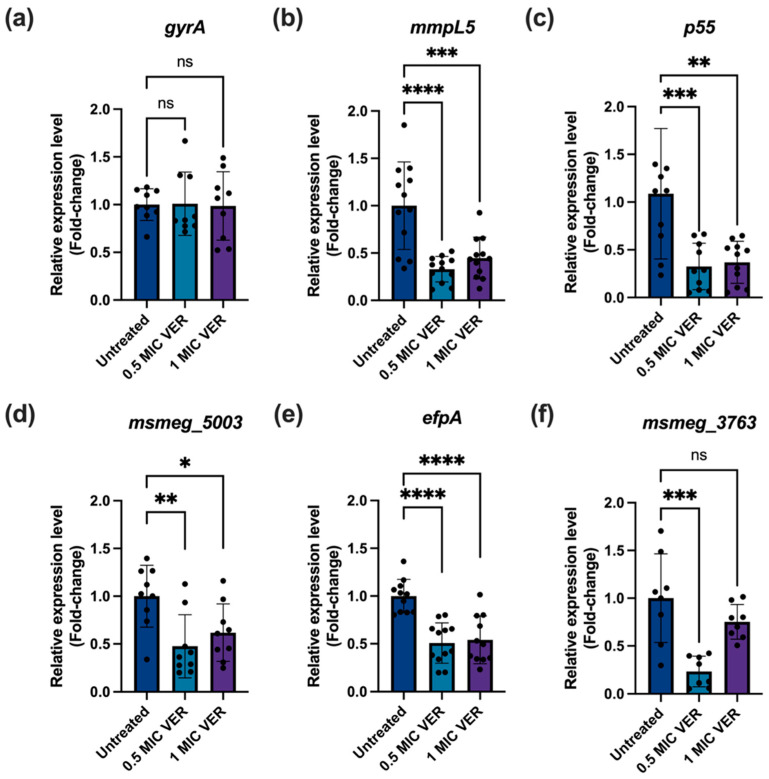
Verapamil decreases efflux pump expression in *M. smegmatis*. The bacteria were treated with verapamil at the indicated concentrations for 48 h and, subsequently, mRNA levels were quantified by qPCR, normalized to *sigA* as a reference gene. (**a**) Expression of the *gyrA* gene as control. (**b**–**f**) The relative expression levels of the selected efflux pump genes: *mmpL5* (*msmeg_1382*), *p55* (*msmeg_3069*), *Tap locus* (*msmeg_5003*), *efpA* (*msmeg_2619*), and *msmeg_3763*, respectively. Three biological replicates were used for each sample, data represent mean ± SD, and significance was shown for comparison to the untreated sample as tested by a one-way ANOVA with Dunnett test for multiple comparisons (ns: not significant, *: *p* <  0.05, **: *p* <  0.01, ***: *p* <  0.001, ****: *p* <  0.0001).

Suppression of permanent genetic resistance upon downregulation of efflux pumps in *E. coli* was mediated by increased expression of DNA repair genes, in particular, the DNA mismatch repair gene *mutS* [18]. We therefore evaluated the impact of verapamil on expression levels of genes encoding efflux pumps and DNA repair enzymes. First, we assessed if the suppression of drug resistance by verapamil correlates with the downregulation of genes encoding efflux pumps. A previous transcriptomic analysis of *M. tuberculosis* H37Rv showed that verapamil reduced the expression levels of most efflux pumps from the Major Facilitator Superfamily (MFS) [39]. We selected several key membrane transporters from different classes in *M. smegmatis* which, based on available experimental data, likely work as efflux pumps and can contribute to drug resistance: MmpL5 from the Resistance-Nodulation-Division (RND) transporter family [4], Tap [40], P55 [10,15] and EfpA [33,41] from the Major Facilitator (MFS) family, and Msmeg_3763 [42,43] from the ATP-binding cassette (ATP) family. Using qPCR, with the DNA gyrase gene A (*gyrA*) as a stable expression control, we observed that the expression levels of all five selected genes encoding components of the efflux pump systems mentioned above were significantly reduced after verapamil pretreatment for 48 h (Figure 5). These results show that verapamil can interfere with expression of efflux pump systems at the transcriptional level.

**Figure 6:**
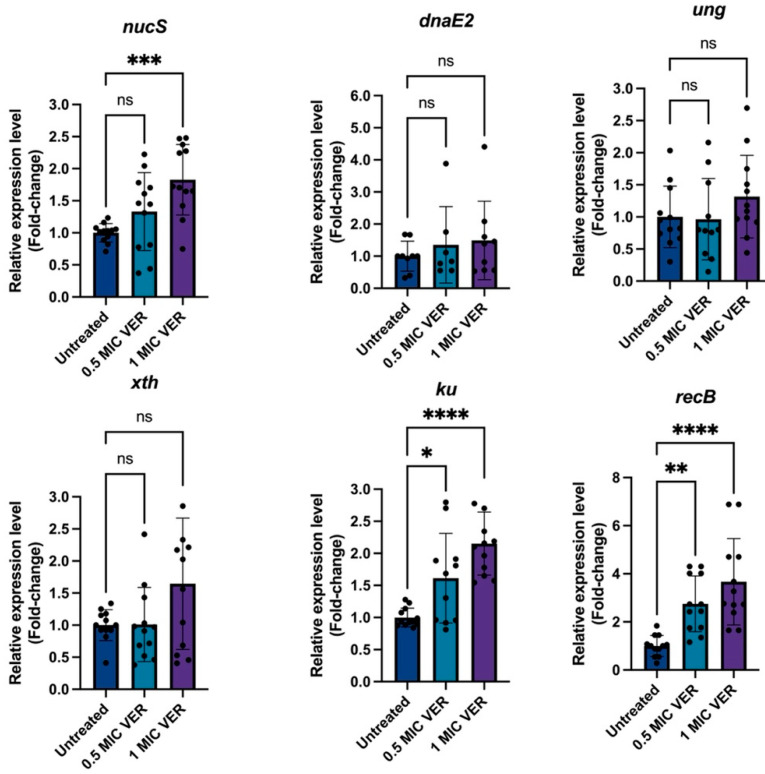
Verapamil upregulates DNA repair gene expression. Quantitative PCR analysis of the relative expression levels of DNA repair genes from different functional classes in untreated and verapamil-pretreated samples at 24 h. Gene expression was normalized to *sigA*, and fold changes were calculated using the 2^−ΔΔCT^ method. Expression in verapamil-pretreated samples was compared to untreated controls. Three biological replicates were used for each sample, data represent mean ± SD. Statistical significance was assessed using one-way ANOVA followed by Dunnett’s multiple comparisons test (ns, not significant; *: *p* <  0.05, **: *p* <  0.01, ***: *p* <  0.001, ****: *p* <  0.0001).

Next, we tested if verapamil could modulate DNA repair mechanisms. Mycobacteria lack the MutS/MutL mismatch repair system of *E. coli*; instead, NucS functions as the primary mismatch repair protein regulating mutation rates and, thereby, resistance levels [44,45,46,47]. Therefore, we employed qPCR to assess if the decreased resistance frequency observed upon treatment with verapamil correlates with the enhanced expression level of *nucS*. While the presence of verapamil did not change *nucS* levels after 48 h (Figure S5), we found significant, 1.83-fold *nucS* induction after 24 h of treatment with verapamil at 1 × MIC (Figure 6). Next to *nucS*, genes encoding the double strand repair proteins Ku and RecB [48] were both induced in the presence of verapamil (Figure 6). Other investigated DNA repair system genes, such as *dnaE2*, *ung*, and *xth* remained unaffected by verapamil treatment. Taken together, treatment with verapamil, at concentrations that decrease the resistance frequency to antibacterials, indeed triggers downregulation of efflux pumps and induces the expression of several DNA repair genes in *M. smegmatis*.

## 3. Discussion

Resistance to antimycobacterial agents is a key factor limiting the efficacy of tuberculosis chemotherapy. Resistance can be due to mutations in the bacterial genome, which typically interfere with drug binding to the target. Alternatively, drug efflux mechanisms can contribute to resistance, as reported for, e.g., bedaquiline [49], moxifloxacin [33], and isoniazid [50]. Our results reveal that verapamil triggers downregulation of efflux pumps and decreases the resistance frequency for a range of antimycobacterial agents. This dual-action mechanism may make verapamil exquisitely suitable to reduce the chance for development of resistance during chemotherapy.

Verapamil can potentiate companion drugs in combination therapy [3,4,5] and enhances the intracellular levels of marker molecules such as ethidium bromide [11,12,13]. Therefore, it is thought that verapamil interferes with small-molecule efflux, although alternative mechanisms have been proposed as well [9,10]. Inhibition of efflux by verapamil may be direct, by specific binding to a cellular target, or indirect, e.g., by dissipating the proton motive force across the bacterial cell membrane. Dissipation of the proton motive force would drain the bacterial energy reserves needed for drug extrusion and therefore inactivate efflux pumps [51,52]. It has indeed been found that verapamil and a recently synthesized verapamil analog, in which the isopropyl group was replaced by an n-octyl group, decreased the proton motive force [53,54], but another study did not confirm this result for verapamil [55]. On the other hand, the structure of a MATE-type efflux pump from *Bacillus halodurans* [56] complexed with verapamil demonstrates that verapamil can directly bind and likely inhibit an efflux pump. Next to inhibition by direct binding to an efflux pump or potential indirect inhibition by decreasing the proton motive force, here we found that verapamil significantly downregulates the expression of various efflux pump systems in *M. smegmatis*. This observed transcriptional downregulation of efflux pumps suggests an additional mechanism by which verapamil may interfere with efflux pump functionality.

Previous studies demonstrated that, in *E. coli*, diminished levels of the AcrB efflux pump correlated with fewer spontaneous mutations and decreased resistance to antibacterials [18,19]. This effect was mediated by the enhanced expression of DNA repair genes [18,19] and by a multitude of intracellular signaling pathways [19]. These results mirror our findings on verapamil, which decreased the expression levels of efflux pumps and increased the levels of various DNA repair proteins. Therefore, we regard it as likely that the decrease in resistance frequency observed for the various antimycobacterial drugs in the presence of verapamil is due to the same mechanism as reported for *E. coli*, although contributions from other mechanisms, e.g., the interference of verapamil with membrane bioenergetics, certainly cannot be excluded.

The observed ability of verapamil to suppress resistance to antibacterials needs to be further explored and, if possible, enhanced. Potent derivatives of verapamil, as recently reported [53,55] or to be synthesized in the future, might allow for further amplifying of the suppression of resistance evolution. Alternatively, compounds like novobiocin and chlorobiocin, which reduced the mutator phenotype in swarming *E. coli* cells [19], might provide valuable input in this regard. Moreover, as our study is limited to in vitro experimentation, it will be important to evaluate if verapamil can suppress the development of resistance against antimycobacterial drugs in in vivo model systems. This evaluation may also be included in clinical trials with human tuberculosis patients using verapamil as an adjuvant agent. As such, verapamil could serve as a critical component in preserving the efficacy of existing antibiotics and potentially extending their clinical lifespan against evolving mycobacterial pathogens.

## 4. Materials and Methods

### 4.1. Strains and Growth Conditions

The *M. smegmatis mc^2^ 155* stock was from our own lab stock (ATCC 700084). *M. avium hominisius* strain 104 was kindly provided by Sanne Peters, Vrije Universiteit Amsterdam [2,36,57]. *M. tuberculosis* mc^2^6230 was a kind gift from Bill Jacobs laboratory, Albert Einstein of College Medicine [37]. *M. smegmatis* mc^2^ 155 and *M. avium hominisius* strain 104 were grown on Middlebrook 7H10 agar plates or in Middlebrook 7H9 broth supplemented with 0.5% (*v*/*v*) glycerol, 0.05% Tween-80 (*v*/*v*), and 10% ADC (NaCl, bovine albumin, dextrose, catalase) at 37 °C, shaken at 150 rpm. For the auxotrophic strain *M. tuberculosis mc^2^6230*, the same procedure was followed, but with 10% OADC (NaCl, bovine albumin, dextrose, catalase, oleic acid) instead of ADC, 0.05% (*v*/*v*) tyloxapol instead of Tween-80, and 0.2% (*v*/*v*) casamino acids and pantothenate (50 μg/mL) were added as described previously [37].

### 4.2. Determination of MIC

The resazurin microtiter assay (REMA) was performed as previously described [58]. *M. smegmatis mc^2^ 155* was grown overnight to OD_600_ = 0.8–1.2 and subsequently diluted with 7H9 + 0.05% Tween-80 medium to OD_600_ = 0.0015. Wells only containing bacterium were set as a positive control. Wells with compounds only in 7H9 + 0.05% Tween-80 medium were set as a negative control. Two-fold serial dilutions of a compound (verapamil, moxifloxacin, streptomycin, rifampicin) were used in the test wells. Following 24 h incubation, the above wells containing 200 µL liquid were supplemented with 25 µL 0.2 mg/mL resazurin dissolved in 7H9 + 0.05% Tween-80 medium. The microtiter plate was then incubated for 2–6 h at 37 °C in the dark. Growth inhibition was measured by a microplate reader at excitation wavelength 530 nm, and emission wavelength 590 nm (FLUOstar Omega, BMG Labtech (Ortenberg, Germany)); the MIC was determined as the lowest drug concentration yielding < 10% of maximal fluorescence.

### 4.3. Resistance Frequency Test

*M. smegmatis* mc^2^ 155 was grown overnight to OD_600_ = 0.8–1.2, centrifuged, and resuspended in fresh medium to OD_600_ = 0.8. For pretreatment, verapamil (from 50 mg/mL stock in H_2_O) was added to a final concentration of 150 μg/mL (0.5 × MIC) or 300 μg/mL (1 × MIC). Concentrations of verapamil > 1 × MIC had too strong an effect on bacterial growth and therefore were not used. The cultures were then incubated for 48 h until reaching the late logarithmic phase. Cells were subsequently washed twice and sonicated twice in a water bath sonicator for 15 s each time to disrupt clumps. The OD_600_ was adjusted to 0.6, followed by 10–20-fold concentration of the bacterial suspension. For drug-containing agar plates, 200 µL of the concentrated bacteria was spread on 7H10 agar containing 2 × MIC moxifloxacin or 2 × MIC streptomycin. For 7H10 agar plates containing 8 × MIC rifampicin, bacteria adjusted to OD_600_ = 0.6 were used for plating. To determine the exact number of viable bacteria in the absence of drugs, 10^7^ or 10^8^-fold dilutions were plated onto drug-free 7H10 + 10% ADC agar plates. Resistance frequency was determined as the ratio of colony-forming units (CFU/mL) obtained on 7H10 agar containing the antibiotic compared to those on drug-free 7H10 agar, adjusted by dilution factor. We employed one-tailed *t*-tests to assess whether verapamil specifically decreases the resistance rate. As our hypothesis is directional—that verapamil treatment will lead to an decrease—a one-tailed test is appropriate to assert this claim.

### 4.4. RNA Isolation, Reverse Transcription and qPCR Analysis

RNA isolation was performed using the NucleoSpin RNA Kit (Machery Nagel (Düren, Germany)). Briefly, *M. smegmatis* wild type with or without verapamil pretreatment was harvested at a density of 10^9^ cells/mL. RNA was isolated according to the manufacturer’s instructions, with the following modifications. The cells were bead-beaten in tubes containing 0.1 mm Zirconium–silica glass beads, 500 μL Buffer RA1, and 5 μL β-mercaptoethanol for 1 min at a speed of 6 m/s for cell lysis. The RNA concentration was determined using a NanoDrop 2000 spectrophotometer (Thermo Fisher (Waltham, MA, USA)). Reverse transcription was performed with a High-Capacity cDNA Reverse Transcription Kit with RNase inhibitor (Applied Biosystems^®^ (Waltham, MA, USA)) according to the manufacturer’s protocol. The obtained cDNA was subjected to qPCR analysis using gene-specific primers (Table S2). The qPCR reaction was performed with a SensiFAST™ SYBR^®^ Hi-ROX Kit (Meridian Bioscience (Cincinnati, OH, USA)) as follows: 95 °C (2 min) followed by 35 cycles of 95 °C (20 s), 60.0 °C (10 s) and 72 °C (20 s). For the quantification of transcriptional changes, we used the comparative 2^−ΔΔCt^ method. The transcript levels of an individual chosen gene were first normalized to *sigma*; subsequently, the fold-change of this ratio for treated samples versus untreated samples was calculated.

### 4.5. DNA Sequencing

*M. smegmatis* colonies resistant to moxifloxacin and non-resistant colonies were picked from the plates by an inoculation loop and stirred in a 1.5 mL Eppendorf tube with 500 μL H_2_O and Zirconia beads (0.1 mm size). The bacteria were lysed by bead-beating for 2.5 min. The tubes were briefly spun down and then incubated at 96 °C for 10 min to allow genomic DNA to be released. Afterward, brief centrifugation was performed to pellet the Zirconia beads and the samples were incubated on ice for 2 min. 1 μL of the supernatant was taken and used as the genomic DNA template for the PCR reaction. The used primer are listed in Table S2. The PCR product was loaded on a 1% agarose gel and run for 20 min at 80 V and purified from the gel using the NucleoSpin PCR clean-up Gel extraction kit (Machery Nagel (Düren, Germany)). The sequencing was performed by Macrogen Europe (Amsterdam, The Netherlands) and sequence alignment was conducted by Snapgene software with version 6.1.1.

### 4.6. Flow Cytometric Analysis of M. smegmatis

*M. smegmatis* was grown to OD_600_ = 0.8–1.2 and pretreated with verapamil for 48 h as described above. Cells were washed twice in 0.9% NaCl + 0.05% Tween-80, adjusted to OD_600_ = 0.6, and diluted 100-fold to 1 × 10^6^ cells/mL. Samples were stained with Vybrant™ DiD (5 μM) (Waltham, MA, USA) [59] and SYTOX orange (100 nM) (Waltham, MA, USA) for 20 min at 37 °C in darkness, washed twice, and analyzed after 10 min using Invitrogen™ Attune™ NxT Flow cytometer (Carlsbad, CA, USA) (DiD: Ex RL1/Em 675/25 nm; SYTOX: Ex YL1/Em 585/40 nm).

## Data Availability

The original contributions presented in this study are included in the article/Supplementary Material. Further inquiries can be directed to the corresponding author(s).

## Supplementary Materials

The following supporting information can be downloaded at: https://www.mdpi.com/article/10.3390/ijms262211124/s1.
