# Supplementary material for: Verapamil Suppresses the Development of Resistance Against Anti-Tuberculosis Drugs in Mycobacteria
Source: Int J Mol Sci. 2025 Nov 17;26(22):11124. doi: 10.3390/ijms262211124 (PMC12652337; doi:10.3390/ijms262211124)
Supplement: Supplementary file 1 [file ijms-26-11124-s001.zip › ijms-3972392-supplementary.pdf]

Supplementary Materials

Verapamil Suppresses the Development of Resistance Against Anti-Tuberculosis Drugs in Mycobacteria

Kunna Liu [1], Elise Buitenhek [1], Coenraad P. Kuijl [2,3], Yuval Mulla [1,*], Joen Luirink [1] and Dirk Bald [1,*]

1 Molecular Microbiology Section, A-LIFE and AIMMS, Faculty of Science, Vrije Universiteit Amsterdam, 1081 HZ Amsterdam, The Netherlands; k.liu@vu.nl (K.L.); s.luirink@vu.nl (J.L.)
2 Medical Microbiology and Infection Control (MMI), Amsterdam University Medical Center Location VUmc, 1081 HZ Amsterdam, The Netherlands; c.kuijl@amsterdamumc.nl
3 Amsterdam Institute for Immunology & Infectious Diseases, Amsterdam University Medical Center Location VUmc, 1081 HZ Amsterdam, The Netherlands
* Correspondence: y.mulla@vu.nl (Y.M.); d.bald@vu.nl (D.B.); Tel.: +31-654-795-478 (D.B.)

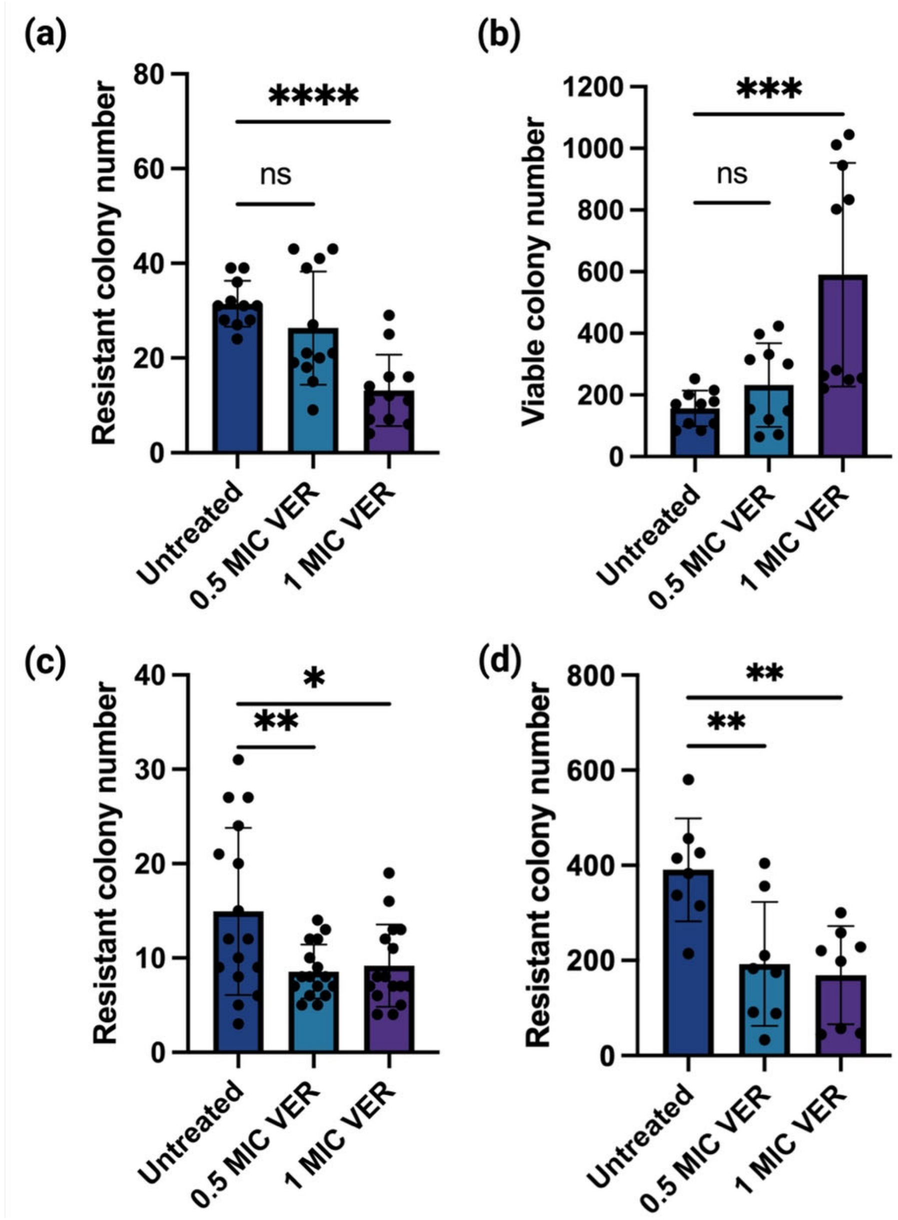

Figure S1. Verapamil pretreatment decreases the number of resistant colonies for moxifloxacin, streptomycin and rifampicin in *M. smegmatis*. (a) Number of resistant colonies growing on agar plates containing 2 × MIC moxifloxacin after verapamil pretreatment. **(b)** The number of viable colonies growing on the antibiotic-free plate in untreated and verapamil-pretreated samples, normalized by OD_{600}. **(c)** Number of resistant colonies growing on agar plates containing 2 × MIC streptomycin after verapamil pretreatment. **(d)** Number of resistant colonies growing on agar plates containing 8x MIC rifampicin. At least three biological replicates were used for each sample and significance was shown for comparison to

the untreated sample, as tested by a one-way ANOVA with Dunnett test for multiple comparisons. Data represent mean ± SD. (ns: not significant, *: $p < 0.05$, **: $p < 0.01$, ***: $p < 0.001$, ****: $p < 0.0001$).

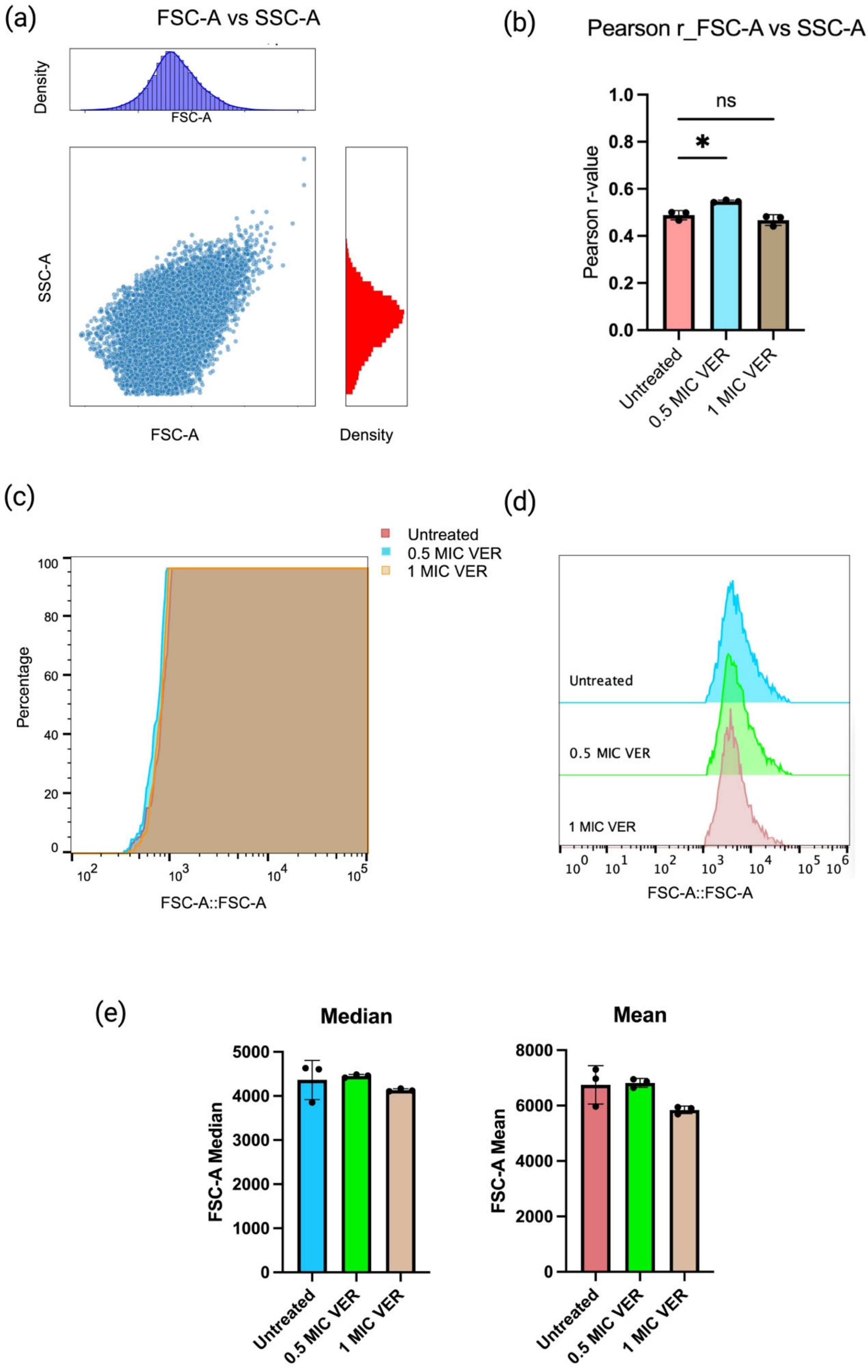

Figure S2. Analysis of bacterial cell size and aggregation by flow cytometry (a) The scatter

plot of FSC-A and SSC-A of *M. smegmatis* and calculated r-value for each read. **(b)** The comparison of the correlation coefficients (r values) between FSC-A and SSC-A across untreated and verapamil (VER) pretreated samples. Three biological replicates were used for each sample and significance was shown for comparison to the untreat sample, as tested by a one-way ANOVA with a Dunnett test (ns: not significant, *: $p < 0.05$). Data represent mean ± SD. **(c)** Overlay of the cumulative distribution function (CDF) for three *M. smegmatis* samples, indicating the population percentage changes along FSC-A changes. **(d)** The histogram distribution of cell size based on the FSC-A value for verapamil (VER) pretreated samples and untreated control. **(e)** The mean and median value of mycobacteria cell size in the whole population based on the FSC-A parameter. Three biological replicates were used for each sample and significance was shown for comparison to the untreat sample, as tested by a one-way ANOVA with a Dunnett test. Data represent mean ± SD.

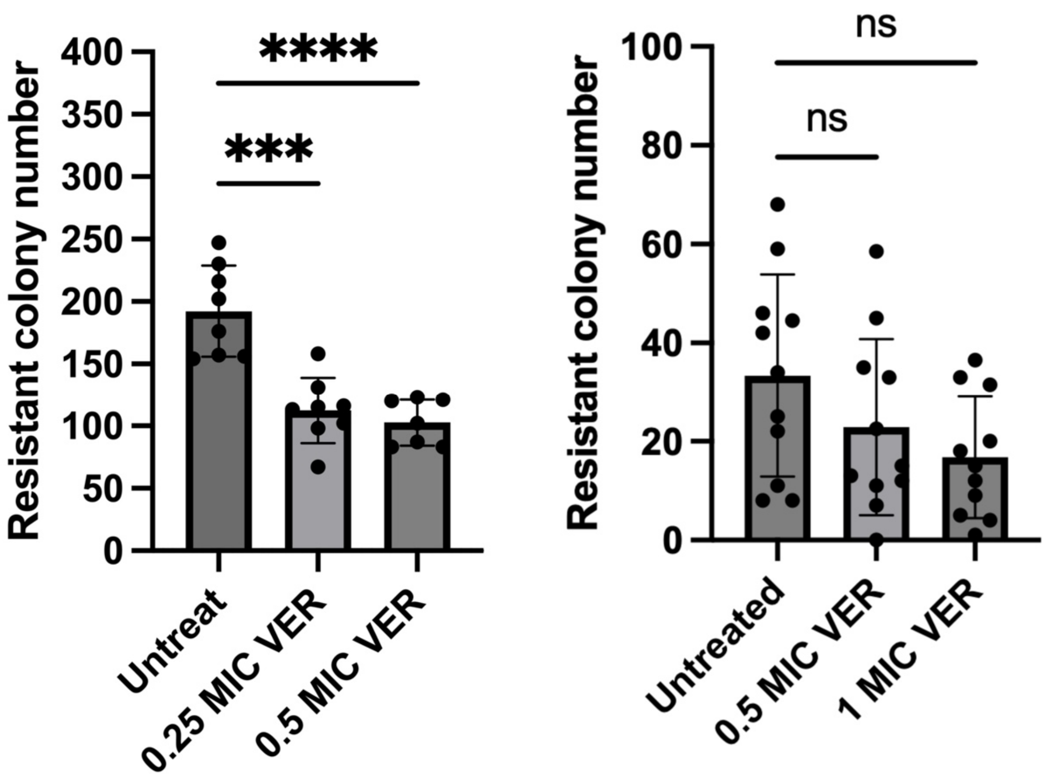

Figure S3. Verapamil pretreatment decreases moxifloxacin-resistant colony number in *M. avium* and *M. tuberculosis*. Number of resistant colonies *M. avium* (left) and *M. tuberculosis* 6230 (right) that grew on agar plates containing 2 × MIC moxifloxacin after verapamil pretreatment. Three biological replicates were used for each sample and significance was shown for comparison to the untreated sample, as tested by a one-way ANOVA with Dunnett test for multiple comparisons. Data represent mean ± SD (ns: not significant, *: $p < 0.05$, **: $p < 0.01$, ***: $p < 0.001$, ****: $p < 0.0001$).

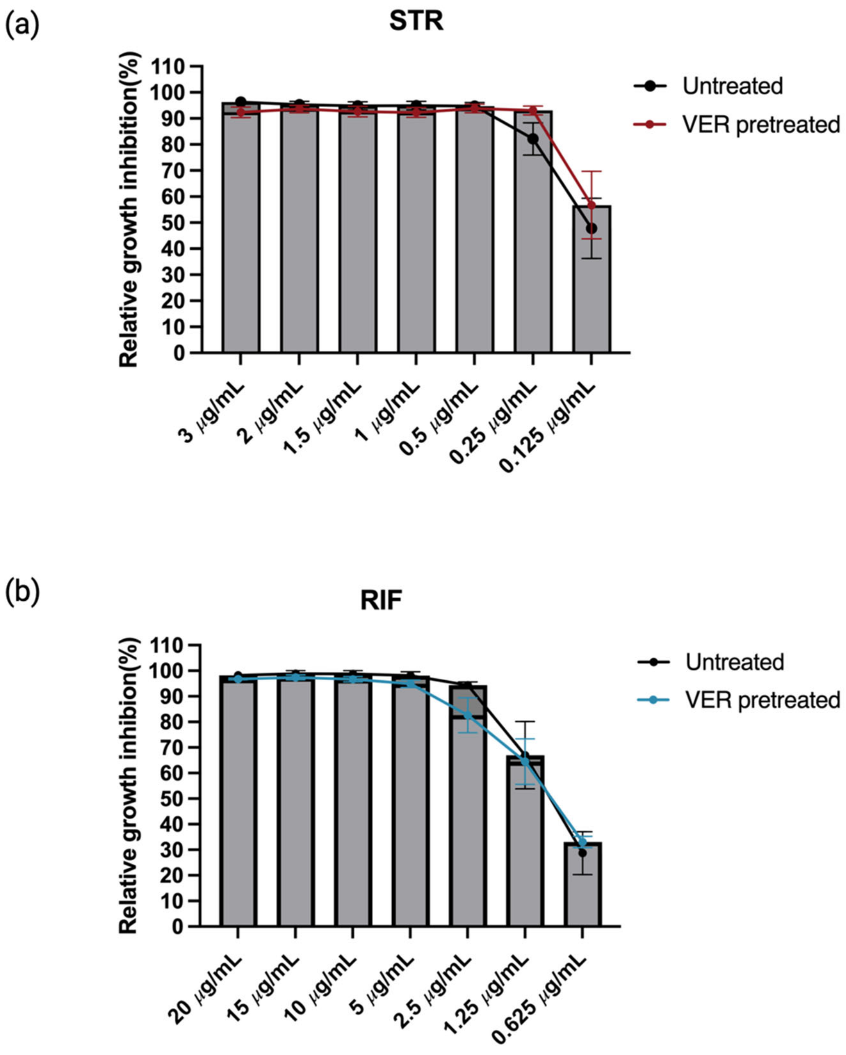

Figure S4. The sensitivity to streptomycin and rifampicin of *M. smegmatis* after pretreatment with verapamil. Growth inhibition of *M. smegmatis* treated with serial concentration dilutions of streptomycin (STR)(**a**) or rifampicin (RIF) (**b**) after pretreatment with verapamil (VER at 1 × MIC) or untreated (same volume of H_2O). Each sample was analyzed using two biological replicates with four technical replicates each, and significance was shown for comparison to the untreated sample, as tested by multiple t-tests. Data represent mean ± SEM (ns: not significant, *: $p < 0.05$, **: $p < 0.01$, ***: $p < 0.001$, ****: $p < 0.0001$).

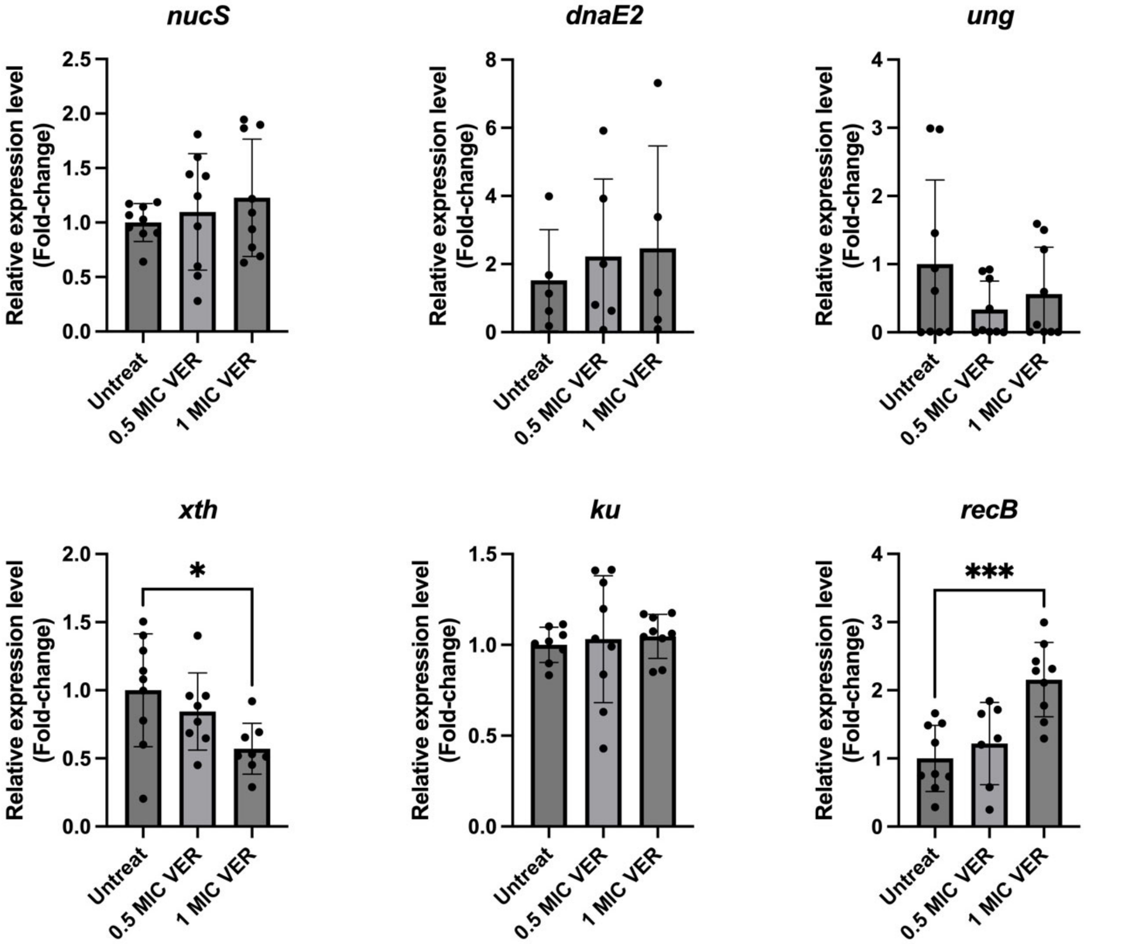

Figure S5. Impact of verapamil on transcription of DNA repair genes. Quantitative PCR analysis of the relative expression levels of DNA repair genes in untreated and verapamil-pretreated samples at 48h. Gene expression was normalized to *sigA*, and fold changes were calculated using the $2^{-\Delta\Delta CT}$ method. Expression levels in verapamil-pretreated samples were compared to untreated controls. Statistical analysis was performed using one-way ANOVA followed by Dunnett's multiple comparisons test and data represent mean ± SD (*: $p < 0.05$, **: $p < 0.01$, ***: $p < 0.001$).

Supplementary Table S1. Resistance frequency of *M.smegmatis mc2 155* against moxifloxacin(MXF), rifampicin(RIF) and streptomycin(STR)

**Resistance frequency of *M.smegmatis mc2 155* against MXF**	
Concentration	Average MXF-resistance frequency(10^{-9})
1.5 MIC MXF	4.40
2 MIC MXF	4.09
3 MIC MXF	1.24
4 MIC MXF	0.30
6 MIC MXF	0.35
8 MIC MXF	0.04
10 MIC MXF	0.16
12 MIC MXF<br>† MXF MIC_{90} = 0.063 µg/mL	0.04
**Resistance frequency of *M.smegmatis mc2 155* against RIF**	
Concentration	Average RIF-resistance frequency(10^{-8})
8 MIC RIF	2.70
10 MIC RIF<br>† RIF MIC_{90} = 5	1.14
**Resistance frequency of *M.smegmatis mc2 155* against STR**	
Concentration	Average RIF-resistance frequency(10^{-9})
1 MIC STR	12.80
2 MIC STR	1.82
4 MIC STR	0.40
6 MIC STR	0.24
8 MIC STR	0.52
10 MIC STR<br>† STR MIC_{90} = 0.5	0.26

Supplementary Table S2. Primers used for qPCR in this research

Primer	Sequence
qMsm_MmpL5-F2	CGTTCTATCTGCCGCCGGAG
qMsm_MmpL5-R2	CTCTTCGGTCAGCGGGTCAC
qMsm_P55-F3	GGAGCCCTGGATGATGCGTC
qMsm_P55-R3	ACGGGGTATCTGCTCGGCTA
qMsm_Rv1258c-F2	GTACGCGGTGCTGTCGAAGT
qMsm_Rv1258c-R2	ACAGCGCCAGGATCAACGG
qMsm_3763-F1	AGTTCCAGGCCGTGCAGTTC
qMsm_3763-R1	AGGATATGCGCCGACCTCCT
qMsm_efpA-F2	GGTGAACCTGGACCGCATCG
qMsm_efpA-R2	CTGCTCGTCGTTCATGGCCT
qMsm_SigA-F2	ATCTCCTTGGCGAGCTCTTC
qMsm_SigA-R2	GGGCTACAAGTTCTCGACCT
qMsm_qgyrA-F1	CCTGCGTGGTCTGGTCAAGG
qMsm_qgyrA-R1	CGATCTTCTGCCGCTCGAGG
qMsm_nucS-F1	ACCTGGAACTGCTCAACCGC
qMsm_nucS-R1	TCCATTCCGCGCATCTGGTC
qMsm_dnaE2-F1	AGGTGATCGAACTGGCCAA
qMsm_dnaE2-R1	TCGAAC TTGACCAGACCGAT
qMsm_Xth-F1	ATCGACTTCATCCTCGGGTC
qMsm_Xth-R1	CAGTTCAGTTCGACGAGCAC
qMsm_Ku-F1	TTCAAACCCGACCTGTACCA
qMsm_Ku-R1	CTTTGAGTCCGACTTGCCAC
qMsm_RecB-F1	ATCTCGGTGGTGTGCTGTAT
qMsm_RecB-R1	GACAATGCGACGACCAGATC
qMsm_UNG-F2	CTCGTCGAGGAAGGTTGGGC
qMsm_UNG-R2	CGACGATCAGCAC CCTCACC
